# Differential Impacts of Fronto‐Cerebellar tACS and tVNS on Motor Learning: Evidence for Sex‐Related Neural Modulation

**DOI:** 10.1155/np/3295986

**Published:** 2026-09-12

**Authors:** Viola Oldrati, Marco Nave, Sara Boscarol, Andrea Ciricugno, Maria Arioli, Zaira Cattaneo, Cosimo Urgesi, Alessandra Finisguerra

**Affiliations:** ^1^ Scientific Institute, IRCCS E. Medea, Bosisio Parini, Lecco, Italy, operapadrepio.it; ^2^ Scientific Institute, IRCCS E. Medea, Pasian di Prato, Udine, Italy, operapadrepio.it; ^3^ Center for Neuroscience, University of Camerino, Camerino, Italy, unicam.it; ^4^ IRCCS C. Mondino Foundation, Pavia, Italy, mondino.it; ^5^ Department of Human and Social Sciences, University of Bergamo, Bergamo, Italy, unibg.it; ^6^ Department of Human and Social Sciences, Universitas Mercatorum, Rome, Italy

**Keywords:** motor learning, noninvasive brain stimulation, sex, transcranial alternating current stimulation, transcutaneous vagus nerve stimulation

## Abstract

Noninvasive brain stimulation (NIBS) techniques rely on distinct mechanisms and engage different neural circuits. For instance, transcranial alternating current stimulation (tACS) targets cortical networks directly, while transcutaneous vagus nerve stimulation (tVNS) influences the brain via peripheral input, reflecting top‐down versus bottom‐up plasticity pathways. Despite their potentialities in modulating plasticity, reports of intra and inter‐subjects variability hamper their reliability as therapeutic approaches for neurorehabilitation. Here, we tested whether sex can represent a possible source of variability. We examined whether tACS and tVNS protocols affect motor learning and explored sex differences among 96 healthy adults, employing a sham‐controlled, between‐subjects design. A total of 48 participants (24 females) received fronto‐cerebellar tACS at an individualized gamma frequency (30–80 Hz), while 48 participants (24 females) underwent tVNS applied to the cymba conchae. Both stimulations were delivered during the performance of a unimanual visuomotor task. Participants used their right index finger to click and drag an object from the center of the screen to a target bin. Analyses were conducted on precision error (PE), the Euclidean distance between the target bin and drop point, and readiness, measured by reaction times (RTs) from paper appearance to click‐to‐collect. Results showed an influence of sex on tACS effects on PE: on average, female participants improved precision for near targets, whereas male participants improved for far targets, with substantial overlap between groups. This was accompanied by a slowing of RTs in both sexes. tVNS had no significant effect on PE for either sex but showed distinct effects on readiness: slowing RTs in the male participants and speeding them in the female participants. The results highlight the need to consider sex as a critical factor modulating the effects of NIBS and explore the underlying cognitive, neural, and physiological mechanisms.

## 1. Introduction

Over the past decade, the use of noninvasive brain stimulation (NIBS) techniques has grown significantly as a means to modulate cortical excitability, cognition, and behavior. This rise in popularity is partly due to their favorable safety profile, cost‐effectiveness, portability, and ease of use [1]. While NIBS share common goals—namely, investigating brain functions in both health and disease [2] and offering potential therapeutic options [3]—they operate through distinct mechanical principles and physiological pathways. A distinction among these techniques can be made based on how they engage the neural circuits. Transcranial stimulation techniques, such as transcranial alternating current stimulation (tACS), directly target cortical networks, while others, like transcutaneous vagus nerve stimulation (tVNS), activate brain regions via peripheral input, thus differentially modulating neural plasticity through top‐down and bottom‐up mechanisms, respectively. The present study is focused on these two techniques.

By means of electrodes placed over the scalp, tACS applies weak electrical currents at specific frequencies to modulate the neuronal activity of target brain regions [4]. Early theories proposed that tACS modulates brain functions by entraining neural populations and aligning brain oscillations with the external current [5]. However, recent evidence indicates that it does not shift the oscillation frequency unless matched to the brain’s endogenous rhythm [6], suggesting that tACS may primarily enhance, rather than override, intrinsic network dynamics. Importantly, this also points to a crucial feature of tACS and generally of NIBS, whose effects are state‐dependent. The effects of brain stimulation are indeed modulated by the ongoing activity and brain state, in terms of brain oscillations, ongoing tasks, and affective or hormonal states. This could also apply to tVNS.

tVNS functions by delivering mild electrical impulses to the vagus nerve through the skin, typically at the ear, where sensory afferent fibers of the thickly myelinated auricular branch of the vagus nerve can be found. Through this bottom‐up pathway, tVNS activates the vagus nerve nuclei in the brainstem, particularly the Nucleus of the Solitary Tract and the Locus Coeruleus (LC) [7, 8], influencing the production of various neurotransmitter systems, including the Norepinephrine‐ (NE) and the γ‐aminobutyric acid‐ (GABA) systems [9], which are essential for synaptic transmission, neuronal excitability, and neuroplasticity [10].

Despite their practical advantages, NIBS techniques face challenges with inconsistent results and substantial inter‐study variability [11, 12]. With respect to tACS and tVNS, part of this heterogeneity is likely due to the incomplete understanding of the neurophysiological mechanisms underpinning these techniques, as well as methodological differences in stimulation parameters between studies employing tACS [6] or tVNS [9, 13]. Differences in the state of brain activation could also help explain discrepancies among studies. Indeed, inducing brain activation, such as physical activity or motor pre‐activation [14, 15], before, during, or immediately after NIBS can modulate brain plasticity. Another significant portion of heterogeneity may arise from intra‐studies variability, namely, the inter‐individual differences in response to the techniques. Among the stable individual traits that can influence the effects of various NIBS techniques—such as anatomical and morphological characteristics [16], age [17, 18], and genetic profile [19, 20]—sex has received relatively little attention [9, 21]. Yet, there is evidence of significant sex differences in key processes involved in cortical plasticity [22–24], highlighting the role of sex in influencing NIBS‐induced plasticity [25, 26].

Differences in tACS effects between female and male participants may be related to the influence of sex hormones on brain oscillations. Notably, brain oscillations have been shown to fluctuate throughout the menstrual cycle, with most evidence focusing on variations in the alpha‐frequency range [27–29]. In a similar vein, the effects of tACS on brain oscillations in the alpha range were shown to vary according to the menstrual cycle in women diagnosed with premenstrual dysphoric disorder [30], in keeping with the state‐dependent effects of tACS [31]. A few other studies have compared the potential differences in tACS effects between male and female participants. Among these, a study applying tACS at a gamma frequency over the dorsolateral prefrontal cortex found a potential trend toward greater improvements in memory performance in male participants compared to female participants [32]. Krebs and colleagues [33] found that theta tACS applied over the prefrontal cortex during a cognitive training showed a trend toward improved performance in younger elderly women, whereas older men benefited more from transcranial direct current stimulation. This aligned with previous research showing that increasing individual alpha frequency with tACS had a positive effect on visuo‐spatial reasoning performance in the female participants but a detrimental effect on performance in the male participants [34]. In contrast, Kasten and colleagues found no evidence of an interaction effect between participants’ sex and tACS, delivered at the individual alpha frequency, on the mental rotation performance [35].

Research on the differential effects of tVNS male and female subjects remains limited. However, it has been suggested that female participants may be more sensitive to tVNS due to sex differences in LC–NE activation, as evidenced by animal studies (see [36] for a review). Moreover, estrogen receptors have been found in the nodose ganglia and nucleus tractus solitarius, areas innervated by the vagus nerve [37]. A recent study found that tVNS enhanced GABA‐Aergic inhibition to a greater extent in female participants than in male participants [38]. Notably, progesterone—present in all individuals, though typically at higher circulating levels in women—modulates neuronal excitability by influencing ion channels, with progesterone’s neuroactive metabolites likely reducing excitability and raising the seizure threshold via binding to the GABA‐A receptor [39]. Additionally, since autonomic nervous system activity is associated with disorders related to menstrual cycle dysfunctions [40] and sex differences have been observed in the autonomic control of the heart [41], functional sex differences in vagal afferent transmission have been suggested [42, 43]. Accordingly, Yokota and colleagues found sex differences in tVNS effects on heart parasympathetic activity, which varied with stimulation intensity [42]. Similarly, De Couck et al. [44] reported a greater increase in a time‐domain measure of heart rate (HR) variability, another index of parasympathetic activity, in female participants compared to male ones after tVNS. Nevertheless, previous clinical studies have not detected sex as a moderating factor of the effects of the invasive stimulation of the cervical branch of the vagus nerve on motor recovery on seizure control [45] and motor recovery [46].

Taken together, while the available evidence is somewhat limited, it suggests that biological sex may modulate the effects of NIBS techniques, potentially accounting for some of the variability in the findings. Unveiling possible sex differences in the response to different NIBS is crucial for fine‐tuning their clinical application for the treatment of motor and cognitive disorders.

In this study, we aimed to explore, in a sample of healthy adults, potential sex differences^1^ in the effects of two stimulation protocols—one utilizing tACS and the other tVNS—on the performance of a task that has been shown to be modulated by both techniques, that is, a unimanual visuomotor task. The study is indeed a follow‐up analysis of behavioral data from different randomized, within‐subject, sham‐controlled studies investigating the effects of either tACS [47] or tVNS [48] (also Boscarol et al., in preparation) on cortical excitability and plasticity. The performance of the visuomotor task has been taken as a behavioral proxy of motor plasticity. Although the original studies recorded different neurophysiological outcome measures, they shared the NIBS protocols and the same task to evaluate the effects of tACS or tVNS on the visuomotor performance. We capitalized on these different datasets and combined and pre‐processed the behavioral data for using analysis methods specifically aimed at investigating sex‐related differences in NIBS effects on behavioral performance.

The tACS was delivered through a fronto‐cerebellar montage, targeting frequencies within the gamma range (30–80 Hz) and specifically tailored to each participant’s individual gamma frequency (IGF). The choice of this frequency range was driven by evidence of the proposed role of gamma in motor control and movement execution (for a review, see [49]). Regarding the choice of the tACS target region, the cerebellum has become a key target for NIBS due to its involvement in both motor and nonmotor functions [50] and its broad connectivity with cortical and subcortical areas, particularly through the dentate‐thalamus‐cortical pathway [51]. Recent studies have investigated the effects of gamma‐frequency tACS over the cerebellum on motor function, given its central role in motor learning and alignment with basal Purkinje cell activity [52]. Several findings suggest that cerebellar gamma tACS can enhance motor cortex excitability, improve reaction times (RTs), and facilitate motor sequence learning [53–57]. However, other studies reported no significant effects on motor performance or corticospinal excitability [58, 59], highlighting the need for further research.

Moreover, the cerebellum’s “closed‐loop” connections with the prefrontal cortex are thought to underlie the integration of sensorimotor information [60]. Therefore, we hypothesized that a fronto‐cerebellar tACS montage could influence long‐range connections between these regions and maximize the effects on visuomotor performance.

Regarding the tVNS protocol, we hypothesized that it could influence visuomotor performance based on both basic and clinical researches. Recent experimental research found that tVNS is able to modulate different motor outcomes, such as motor planning [61] and adaptation [62]. Moreover, animal models showed that pairing VNS with motor activity can drive cortical reorganization and enhance recovery through specific forms of cortical plasticity [63]. Accordingly, promising results have been obtained in improving motor function by tVNS delivery after stroke in adults [64, 65].

In terms of sex differences, building on prior research suggesting that tACS effects may differ between males and females [32–35], we examined whether our setup and stimulation parameters produced sex‐related differences. Furthermore, tVNS was expected to have a greater effect in women than in men, considering their greater autonomic reactivity after tVNS [44].

## 2. Methods

### 2.1. Participants

We collected the behavioral data of 96 healthy participants. For the tACS group, there were 48 participants (24 females; mean age = 24.35 ± 3.93 SD, range: 20–34 years old), and for the tVNS group, there were 48 participants (24 females; mean age = 23.38 ± 4.01 SD, range: 18–37 years old). The tACS group included the 44 participants tested in Ciricugno et al. [47] and four more participants recruited ad hoc for this study to balance the sample size and sex distribution of the two groups. The tVNS group comprised the 22 participants performing the visuo‐motor task with the right hand in Boscarol et al. [48] and 26 participants that performed the same task with the right hand in a further experiment (Boscarol et al., in preparation). A 2 × 2 ANOVA on age with group (tACS vs. tVNS) and sex (females vs. males) as between‐subject variables revealed no significant effects (all Fs < 4, ps > 0.085), indicating that participants’ age was comparable across groups and sexes.

Before the experimental procedure, all participants, except for three females in the tVNS group, completed the questionnaire by Giustiniani and colleagues [53], comprising also questions about the assumption of contraceptives and, for women, the date of the onset of their last menstrual cycle. No difference was detected between the tACS and tVNS groups in percentage of female participants using hormonal contraceptives (66.7% vs. 62.5%; Fisher’s Exact Test: *p* = 1.00, odds ratio = 1.20), or in the number of days between the last menstrual cycle and the stimulation session (tACS, 12.04 ± 8.65 vs. tVNS, 17.24 ± 12.14; *t* = 1.63, *p* = 0.11).

After providing an overview of the study, including general information about the used NIBS, all participants, who remained naïve to the experimental hypothesis and to the conditions of stimulation throughout the whole experimental sessions, gave written informed consent for participation. The study was approved by the local ethics committees (Comitato Etico IRCCS. Eugenio Medea Prot. N. 66/22 and Comitato Etico IRCCS Policlinico San Matteo Foundation Prot. N. 0014,336/23) and conducted in accordance with the Declaration of Helsinki. Prior to the experiment, the participants were screened to evaluate their suitability for the application of NIBS [9, 66]. None of the volunteers had a history of neurological disorders, brain trauma, or a family history of epilepsy or cardiac problems. Furthermore, they were free from medication, had normal or corrected‐to‐normal vision, and were right‐handed, as confirmed by the Standard Handedness Inventory [67]. Only after completing the whole experiment, participants received information about the experimental hypothesis.

### 2.2. Visuomotor Task

For both the tVNS and tACS experiments, participants completed a custom computer‐based visuomotor task designed to assess manual visuomotor control while undergoing the stimulation protocol—see [47, 48, 68]. This task was programed using E‐Prime 3 software (Psychology Software Tools, Inc., Pittsburgh, PA, USA) and displayed on a 17‐inch monitor with a 60 Hz refresh rate and 1280 x 768 resolution.

**Figure 1:**
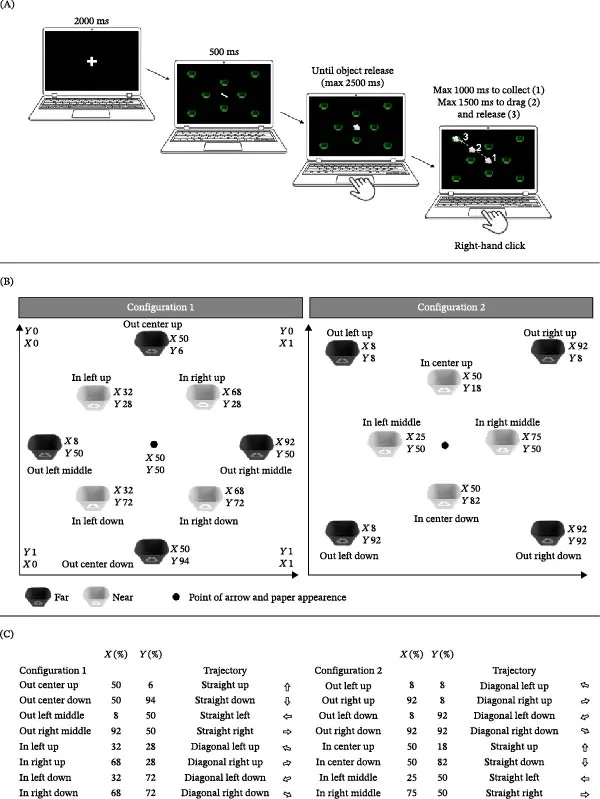
(A) Example of a single trial in the visuomotor task. (B) Illustration of the two visuomotor task configurations, each showing eight recycling bins: four located at a “near” distance from the center of the screen (in light gray) and four at a “far” distance (in dark gray). Note: In the actual task all bins were displayed in green, the grayscale is used to represent the distance of the bins. (C) Schematic description of the recycling bin positions, calculated as percentage‐based proportions of screen size with the origin at the default top‐left corner.

Each trial began with a 2000 ms fixation cross, followed by a 500 ms directional arrow. A crumpled paper object then appeared at the center of the screen. The participants’ objective was to click and drag this object to a target recycling bin, which was situated within a configuration of other bins, following the direction indicated by the arrow. Participants used only their right index finger for the click‐and‐drag operation via a touchpad. The participants had 1000 ms to click and collect the object. The entire drag‐and‐drop operation, from the initial click to release, had to be completed within 1500 ms, after which the object would disappear. The total click‐and‐drag operation, starting from the object’s appearance, needed to be completed within 2500 ms (see Figure 1A).

Two distinct configurations were created for the recycling bins, each displaying eight bins. Four bins were positioned “far” from the screen center, making them more challenging to target, while the other four bins were positioned “near” the center, making them easier. Both far and near targets could be reached via distinct trajectories: diagonal or straight movements (see Figure 1B). Each configuration accounted for 50% of the trials, and their presentation was randomized within blocks (i.e., 40 trials per configuration per block). Participants completed four blocks of 80 trials each during real or sham NIBS administration, resulting in a total of 320 trials per session, lasting ~20 min.

Trials were categorized based on the completion status. A trial was labeled “valid” if the participant successfully completed the click‐and‐drag operation within the specified time limits. Conversely, if the participant failed to click and collect the paper within the initial 1000 ms, the trial was marked “invalid.” If the drag operation was correctly initiated but the participant failed to release the paper within 1500 ms, the program recorded the paper’s final position before starting a new trial; these were specifically labeled as “overtime” trials.

### 2.3. Behavioral Measures Definition

We extracted two key metrics from participants’ task performance: precision error (PE) and RT. PE was calculated as the Euclidean distance (in pixels) between the paper’s final drop position and the center of the cued target bin. To compute PE, since the *x* and *y* coordinates were expressed in % of screen size, with the origin at the top‐left corner (see Figure 1C), we converted the *x* and *y* coordinates of both the bins (binx and bin*y*) and the final drop position (dropx and dropy) into pixel values, based on the screen dimensions. For example, if an *x*‐coordinate was recorded as 7% of the screen, its pixel position was calculated as (7/100) × 1280 = 89.6 pixels). The Euclidean distance, *d*, between the target bin and the paper’s final drop position for each trial was calculated using the formula:

d=√binx – dropx22+biny – dropy.

A lower distance (i.e., values approaching 0) indicates a more precise release operation.

RT was defined as the interval (in milliseconds) from the moment the paper appeared on the screen to the participant’s click‐to‐collect operation. This metric served as an assessment of response readiness.

The administered visuomotor task aimed to probe distinct components of performance: a motor component, as assessed by PE, and a visual attention component—encompassing the orientation toward the target following the directional cue and the readiness to initiate movement (time‐to‐click). The inclusion of time‐to‐click is theoretically motivated by the evidence from both stimulation techniques examined in this study. Cerebellar tACS has been shown to slow down RT in learned but not novel sequences [53], to improve RTs for practiced sequences following stimulation [54], and to accelerate RT gains during early stages of sequence learning [69], establishing time‐to‐click RT as a sensitive index of stimulation‐induced changes in motor learning. Complementarily, tVNS has documented effects on attentional processing [70, 71] and on response initiation latency prior to movement execution [72]. Time‐to‐click therefore constitutes a measure sensitive to early‐stage engagement and initiation processes that tACS and tVNS may modulate through distinct mechanisms.

### 2.4. General Procedure

**Figure 2:**
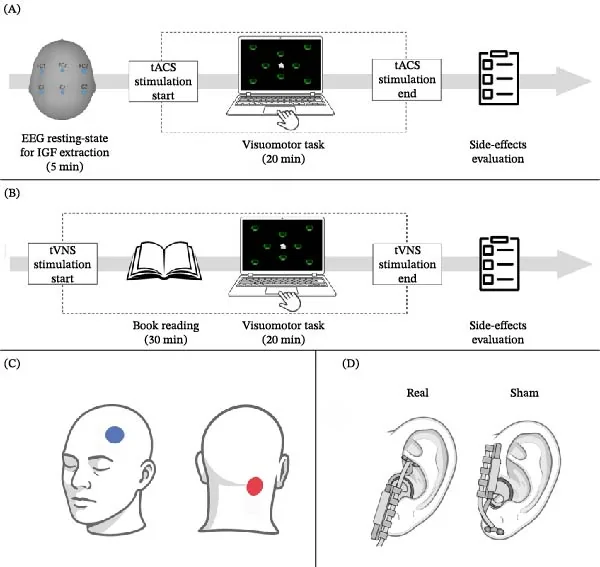
Schematic depiction of the experimental procedures for (A) the tACS group and (B) tVNS group. The stimulation period (real or sham, depending on the session) is indicated by the dashed box. Please note that in the tACS sham condition, the stimulation was turned off after 30 s, while the electrodes remained in place for the duration of the task to maintain participant blinding to the stimulation condition. The EEG resting state recording was performed for IGF extraction. (C) illustrates tACS electrode positioning over the scalp (blue electrode on F3 and red electrode on the right cerebellum). (D) illustrates the electrode placement for tVNS according to the stimulation condition.

Participants in the tACS experiment received either real or sham tACS, administered across two separate sessions (see Figure 2A). At the start of the first session, 5 min of eyes‐open resting‐state EEG activity was recorded. This recording was used to identify each participant’s individual peak frequency (IGF) within the gamma band (30–80 Hz) that exhibited the greatest power during rest; this IGF was then used as the tailored stimulation frequency (detailed EEG recording and preprocessing information are available in the Supporting Information). Following this, participants performed a 5 min version of the visuomotor task during EEG recordings (EEG results are not reported here as they fall beyond the scope of the study). Subsequently, participants received 20 min of either real or sham tACS at their IGF while performing the visuomotor task until stimulation concluded.

For the tVNS experiment, participants received approximately 1 h of either real or sham tVNS, administered across two separate sessions (see Figure 2B). To ensure sufficient activation of the vagus nerve during the task, participants began the visuomotor task 30 min after the stimulation onset. During these initial 30 min of each session, participants were instructed to read a book to control for their activation in a rest condition and ensure comparability across participants and sessions. They then proceeded to perform the visuomotor task (lasting about 20 min). Given the possible effects of tVNS on cardiovascular activity [9], for participants in the tVNS group, cardiovascular parameters, specifically HR and oxygen saturation (SpO_2_), were monitored before the start of tVNS (T0), 30 min after the start of stimulation (T1), and at the end of the tVNS session (T2) to assess the safety and potential unwanted effects of the stimulation (see Supporting Information for further details and analysis).

The visuomotor task had the same number of trials, duration, and structure in the tACS and tVNS groups. As mentioned, participants in the tACS group, but not those in the tVNS group, performed the visuomotor task during the 5‐min recording before stimulation, thus allowing an extra practice of the task. However, this was repeated in both the active and sham tACS sessions, thus not affecting the sham‐controlled comparison of the effects of the two techniques.

In both the tACS and tVNS experiments, the order of real and sham sessions was counterbalanced across participants. Furthermore, the two sessions were scheduled at least 24 h apart to ensure a sufficient washout period. The average distance between sessions was 10.23 days (SD = 6.65) for the tACS experiment and 3.42 days (SD = 5.17) for the tVNS experiment. Both experiments concluded with the administration of a questionnaire designed to assess stimulation‐induced sensations. Data gathered from the tolerability questionnaire are reported in the Supporting Information.

### 2.5. tACS Setup

tACS was delivered using an eight‐channel multi‐focal tES‐EEG device (Starstim, Neuroelectrics, Barcelona, Spain). Stimulation was applied via two 8 cm^2^ ring electrodes, each inserted into a synthetic saline‐soaked sponge. The active electrode was positioned over the right cerebellar hemisphere, centered 2 cm below and 3 cm to the right of the inion (Iz), and the return electrode was placed over the left prefrontal cortex (F3) (see Figure 2C). Participants received 2 mA of tACS, consisting of sinusoidal currents tuned to their IGF (mean IGF = 53.38 Hz and SD = 17.79). The stimulation included a 30 s ramp‐up and ramp‐down phase, and it was applied for a total of 20 min, covering the entire duration of the visuomotor task. For the sham control condition, currents were similarly ramped up and down for 30 s, after which the stimulation was turned off.

### 2.6. tVNS Setup

tVNS was administered using the tVNS‐E device (tVNS Technologies GmbH). This device consists of a programable stimulation unit connected to two titanium ear electrodes mounted on a gel frame. Before electrode placement, participants cleaned their left external ear with alcohol swabs, and a spray solution was applied to the electrodes to enhance the conductivity. For the real tVNS condition, electrodes were placed over the cymba conchae, while for sham stimulation, electrodes were positioned over the earlobe (see Figure 2D). This approach ensured that both real and sham conditions induced comparable sensations, while only targeting the cymba conchae stimulated the vagus nerve pathway due to its higher density of vagal innervation [73]. The electrodes were placed solely on the left ear. The intensity of tVNS was set to the perceptual threshold for each participant. This was defined as the intensity at which participants first perceived sensations, minus 0.1 mA below the point where they reported discomfort at the electrode site. This method was consistently applied across both active and sham sessions leading to a higher intensity in the sham (1.62 ± 0.64 mA) than in the real stimulation session (1.38 ± 0.55 mA; *t* = 1.97, *p* = 0.052). Stimulation alternated between 30‐s on/off periods with a frequency of 25 Hz and a pulse width of 250 µs.

## 3. Data Handling and Statistical Analysis

Before conducting statistical analyses, invalid trials (2.3%) and overtime trials (2.8%) were removed. Subsequently, data were filtered based on PE using a robust outlier detection method described by [74]. Specifically, we used the median as a robust estimate of central tendency (robust center) and the tau‐estimate as a robust measure of scale (robust scale), as recommended by Maronna and Zamar [75]. Unlike the arithmetic mean and standard deviation, which are sensitive to outliers, these robust statistics provide more reliable estimates in the presence of extreme values. Trials with PE values falling beyond ±3.5 robust scale units from the robust center—calculated within each participant, for each combination of stimulation type, distance, and trajectory—were excluded. This procedure excluded 1.4% of trials from the active stimulation condition and 1.1% from the sham stimulation condition, divided as follows: real tACS (0.59%), sham tACS (0.44%), real tVNS (0.79%), and sham tVNS (0.66%).

We then performed two separate linear mixed‐effects models (LMMs) on PE and RT using the lmer function from the lme4 R package [76]. *F*‐statistics and *p*‐values were obtained using a Type III Analysis of Variance Table with Satterthwaite’s approximation for the degrees of freedom. As a result of a technical problem, one participant in the tACS group lacks data from the real stimulation condition, having only data from the sham condition.

The models included four fixed factors: Group (tACS vs. tVNS), stimulation (real vs. sham), distance (far vs. near target), and sex (female vs. male), along with their interactions. Subjects were included as a random factor with random intercepts. The initial model formula was specified as: lmer(dependent_variable ~ group × stimulation × distance × sex + (1 | Subject)). When significant four‐way interactions were detected, we employed a model‐splitting approach to facilitate the interpretation. Specifically, we decomposed the full model into subsets that focused on the factors driving the high‐order interaction. Post‐hoc analyses were performed to explore two‐ and three‐way significant interactions by applying false discovery rate (FDR) correction. Mean values and ± standard errors are reported for all significant main effects and post hoc comparisons. Standardized regression coefficients (β) were computed to assess the effect sizes.

As an exploratory, within the tVNS group, we additionally related HR—recorded at T0 (before stimulation), T1 (30 min after onset, at task onset), and T2 (end of session)—to per‐session task performance (mean PE and RT), correlating (Pearson) HR levels and within‐session HR change scores with behavior, both within each stimulation condition and for the real/sham contrast, overall and by sex.

Given the convenience nature of the sample, whose size was determined a priori in the original studies, we used the G‐Power 3.1 software [77] to conduct a sensitivity analysis and determine the minimum effect size that we could detect with our sample with a power of 80% and a significance threshold of 0.05. The analysis revealed that, with a sample of 96 participants, we could detect an effect size of at least *f* = 0.144 in our mixed model design (numerator df = 1), assuming a correlation among repeated measure of 0.5.

## 4. Results

### 4.1. PE

The four‐factor model on PE revealed several significant main effects and interactions, as reported in Table S1 (four‐factor model; see Supporting Information). Due to a significant four‐way interaction of group × stimulation × distance × sex, *F* (1, 53,148) = 6.29, *p* = 0.012, β_tvns × sham × near × males_ = 0.17, we conducted separate models for each group.

**Figure 3:**
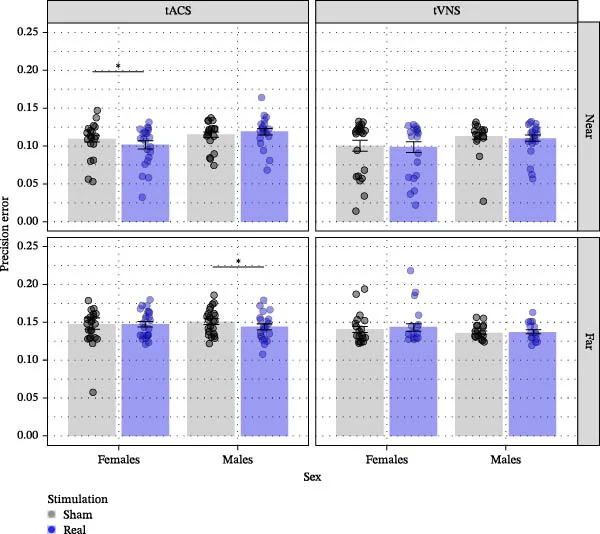
Mean precision error and individual observations across group (tACS vs. tVNS), sex (females vs. males) and target bin distance (far vs. near), color‐coded by stimulation (real vs. sham).  ^∗^ *p*  < 0.05. Error bars represent ±1 SE.

The three‐factor model on the tACS sample yielded a significant main effect of distance, *F* (1, 27032) = 595.52, *p*  < 0.0001, *β*_near_ = −0.36, indicating lower PE in reaching near (0.11 ± 0.003) vs. far (0.15 ± 0.003) target bins. The model showed also a significant interaction of distance × sex, *F* (1, 27032.6) = 11.01, *p* = 0.001, *β*_near × males_ = 0.16, with lower PE in females (0.11 ± 0.004) than males (0.12 ± 0.003; *p* = 0.01) in reaching near target bins but not far target bins (females: 0.15 ± 0.004; males: 0.15 ± 0.003; *p* = 0.87). This interaction was further qualified by a significant three‐way interaction of stimulation × distance × sex, *F* (1, 27031.4) = 10.50, *p* = 0.001, *β*_sham × near × males_ = −0.16 (see Figure 3). Post‐hoc analysis showed that, in females, active tACS reduced PE (0.10 ± 0.005) compared to sham tACS (0.11 ± 0.005; *p* = 0.008) for near target bins, while no such difference emerged for far target bins (active: 0.15 ± 0.004; sham: 0.15 ± 0.007; *p* = 0.84). Conversely, in males, active tACS reduced PE (0.14 ± 0.004) compared to sham tACS (0.15 ± 0.003; *p* = 0.03) for far target bins, but not for near target bins (active: 0.12 ± 0.004; sham: 0.12 ± 0.003; *p* = 0.16). Other effects were not significant, all Fs < 4, ps > 0.06.

The three‐factor model on the tVNS sample yielded a significant main effect of distance, *F* (1, 26125.7) = 391.05, *p* < 0.0001, *β*_near_ = −0.29, indicating lower PE in reaching near (0.11 ± 0.004) vs. far target bins (0.14 ± 0.002). The interaction distance × sex was also significant, *F* (1, 26125.7) = 12.62, *p* = 0.0004, *β*_near × sham_ = 0.08, with lower PE in females (0.10 ± 0.006) than males (0.11 ± 0.004; *p* = 0.003) for near target bins, but not far target bins (females: 0.14 ± 0.004; males: 0.14 ± 0.002; *p* = 0.29). Other effects were not significant, all Fs < 2, ps > 0.19.

### 4.2. RT

**Figure 4:**
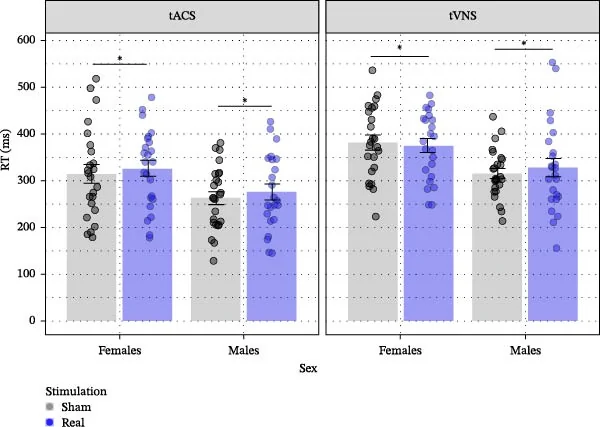
Mean RT (ms) and individual observations across sex (females vs. males) and group (tACS vs. tVNS), color‐coded by stimulation (real vs. sham).  ^∗^ *p*  < 0.05. Error bars represent ±1 SE.

The four‐factor model on RT (in ms) revealed the following significant main effects: Group, *F* (1, 92) = 14.56, *p* = 0.0003, *β*_tVNS_ = 0.32, with faster RT in the tACS group (293.76 ± 11.34) than the tVNS group (349.06 ± 10.39); stimulation, *F* (1, 53,145) = 51.41, *p* < 0.0001, *β*_sham_ = −0.13, with slower RT during real (326.79 ± 9.12) than sham stimulation (318.77 ± 8.74); sex, *F* (1, 92) = 14.09, *p* = 0.0003, *β*_males_ = −0.38, with faster RT in males (294.23 ± 10.42) than females (348.61 ± 11.35). Moreover, the model showed significant interactions of group × stimulation, *F* (1, 53,145) = 41.53, *p*  < 0.0001, *β*_tVNS × sham_ = 0.19, and stimulation × sex, *F* (1, 53145) = 13.27, *p*  < 0.0001, *β*_sham × males_ = 0.05, which were further qualified by a three‐way interaction of group × stimulation × sex, *F* (1, 53145) = 41.38, *p*  < 0.0001, *β*_tVNS × sham × males_ = −0.21 (see Figure 4). Post hoc analysis revealed that real tACS slowed RT compared to sham in both females (real: 326.46 ± 17.03; sham: 314.58 ± 19.80; *p*  < 0.0001) and males (real: 276.13 ± 16.89; sham: 263.25 ± 13.98; *p*  < 0.0001). Differently, real tVNS increased RT (327.75 ± 19.59) compared to sham (315.44 ± 10.93; *p*  < 0.0001) in males but reduced them in females (real: 374.69 ± 14.29; sham: 381.79 ± 15.74; *p*  < 0.0001). No other effects were significant, all Fs < 2, ps > 0.17.

## 5. Discussion

In this study, we explored the sex‐related effects of two different NIBS, namely, tACS and tVNS, on the performance in a visuomotor task. These techniques were applied to two distinct groups of healthy adults, receiving, in two separate studies, either gamma tACS or tVNS, in a within‐subject sham‐controlled design. The main aim was to explore potential sex differences in the effects of the two stimulation protocols. We evaluated performance using two key metrics: a movement precision measure, indicated by the PE, and a time‐based measure of readiness, measured by the RT from paper appearance to click‐to‐collect operation.

### 5.1. Sex Differences in Visuomotor Performance

The model on PE revealed that the performance differed between sexes, independent of the stimulation group. Specifically, on average, female participants demonstrated greater precision than male participants when performing the drag‐and‐drop task toward near target bins—those positioned closer to the center of the screen and requiring a shorter range of movement. However, this sex difference did not emerge when participants aimed for far target bins. This pattern is consistent with reports of, on average, a relative advantage in manual dexterity and fine motor control in the female participants and in aiming tasks in the male participants, although these literature rest in part on developmental samples and are themselves characterized by substantial overlap between groups [78–81], so any extrapolation to the present touchpad task is tentative. We can only speculate that differences in the motor‐control strategy, rather than anatomical or muscular factors (which are unlikely to be engaged by a seated finger‐dragging task [82]), may have contributed to how near versus far targets were processed during goal‐directed movements.

The analysis of RT also revealed a sex difference across all groups: males were generally faster than females in the click‐to‐collect operation. This finding aligns with a body of research demonstrating that males, on average, exhibit faster reaction and shorter movement times across a range of motor tasks. Several studies reported that males outperform females in tasks requiring rapid responses, such as reaching and button‐pressing [83–85]. Similarly, faster RT have been observed in male as compared to female athletes [86, 87]. Meta‐analyses spanning diverse motor performance domains further supported this pattern, showing that males tend to achieve shorter execution times in several motor tasks [88, 89]. These differences may be attributed to a combination of factors, including differences in muscle strength [90], distinct motor strategies [91], and sex‐related variations in neurological processing [92].

Interestingly, the effects of stimulation revealed distinct patterns: real tACS was associated with improved movement precision but also with increased RTs, suggesting a potential trade‐off between speed and accuracy induced by the stimulation in both female and male participants. Additionally, a sex‐related differential effect emerged under the real tVNS. These findings, explored further in the following sections, may rely on sex differences in motor performance, potentially reflecting underlying factors such as divergent motor control strategies and/or neurophysiological mechanisms across sexes.

### 5.2. Sex‐Based Differences in tACS Effects

For the tACS group, overall, the analysis indicated that tACS modulated motor performance, in line with previous research [53–57]. Specifically, fronto‐cerebellar gamma tACS might have improved movement precision, possibly due to its modulation of neural circuits involved in motor prediction [93] and fine‐tuned sensorimotor control [50, 94]. This form of stimulation targets oscillatory communication between the cerebellum and the prefrontal cortex—two regions critically involved in coordinating movement, predicting sensory consequences of motor output in real time [95]. The cerebellum plays a crucial role in forming internal models that anticipate the outcome of actions, allowing for precise timing and error correction [94], while the prefrontal cortex contributes to planning [96] and goal‐directed behavior [97].

Crucially, we found that real tACS improved movement precision in reaching near targets in females and far ones in males, which suggests that the stimulation may have interacted with sex‐related differences in motor planning or motor execution strategies. Indeed, if females rely more on proximal sensorimotor strategies, this could make their performance more susceptible to stimulation during near‐reaching tasks that require local control. On the other hand, males have been shown to exhibit faster visuomotor decision‐making in tasks requiring rapid integration of visual information with motor planning for targets [85]. This may reflect a stronger reliance on feedforward mechanisms and spatial prediction—processes that are potentially more sensitive to neuromodulation during far‐reaching movements. Yet, these interpretations are speculative and warrant further investigation.

Sex‐related differences in hemispheric lateralization and connectivity have been proposed as contributing factors in motor tasks [98], which may affect how tACS, particularly when applied over lateralized fronto‐cerebellar networks, modulates cortical excitability and motor output. Notably, fluctuating sex hormone levels across the menstrual cycle have also been shown to affect functional cerebral asymmetries, likely through the neuromodulatory effects of hormones and their metabolites on interhemispheric processing. Extending these findings to the motor domain, an interesting study found that fine motor coordination and manual asymmetries are similarly susceptible to cycle‐related hormonal fluctuations, presumably due to hormone‐induced modulation of interhemispheric interaction [99]. Taken together, these observations highlight a complex interplay between sex and hemispheric specialization, which may, in turn, influence the neuromodulatory effects of the right cerebellum‐ left frontal cortex tACS protocol applied here.

We also found that real tACS slowed RT in both females and males. Although this effect co‐occurred with improved movement precision, further explorative analyses suggest that tACS may modulate response initiation and motor precision through partially independent mechanisms. Indeed, an exploratory LMM performed to examine the influence of RT on PE in the tACS group revealed a very small yet significant effect of (*F*(1, 8334) = 7.77, *p* = 0.005, *β* = 0.02). The direction of the effect indicates that longer time‐to‐click responses were associated with higher, rather than lower, PE, suggesting that slower responses do not reflect more effective motor preparation but may instead index less efficient trials. This interpretation is consistent with prior work showing that tACS can differentially affect the distinct components of task performance. For example, a meta‐analysis on tACS effects in working memory tasks reported improved accuracy under real stimulation, sometimes accompanied by slower RTs [100] (but see [101] for another meta‐analysis reporting no reliable effect of tACS on RT).

### 5.3. Sex‐Based Differences in tVNS Effects

The findings on the impact of tVNS revealed a null effect on movement precision and a sex‐related effect on RT. First, the absence of a significant effect on performance accuracy misaligns with the results of recent research that found tVNS effects on different motor outcomes, such as motor planning [61] and adaptation [62]. Yet, differences in stimulation parameters—such as targeting the tragus and using shorter (1.5 s) phasic bursts at higher frequency (30 Hz) and lower pulse width (100 µs), compared to targeting the cymba conchae and to longer (30 s on/off) trains at lower frequency (25 Hz) and higher pulse width (250 µs) used here—as well as differences in the task, probing either end‐state comfort [61] or finger force modulation [62], may account for the observed discrepancies.

For what concerns the time metric, differently from tACS, tVNS effects on RT were sex‐related. Previous research on tVNS has reported mixed effects on RT across various behavioral paradigms, some studies have found increases [102], others decreases [103], and still others no significant effects [104], highlighting the influence of methodological differences. However, to our knowledge, no prior studies have examined whether these effects differ by sex.

Our finding of a sex‐related RT effect may be understood in the context of emerging literature on sex differences in vagally mediated neural circuits. A relevant study [105] found that higher vagally mediated HR variability was associated with fewer very slow RTs, suggesting reduced attentional lapses and enhanced cognitive readiness. This aligns with prior evidence linking cardiac vagal control to goal‐directed attention [106]. While Spangler et al. [105] did not explicitly assess sex differences, their predominantly female sample (72%) might partly account for the observed effects. Although speculative, it is plausible that sex‐related changes in parasympathetic activity contribute to the differential impact of tVNS on the behavioral readiness observed in our study.

On the same line, a potential explanation for the observed sex differences in tVNS‐induced behavioral effects relates to structural and functional differences in the LC, a brainstem nucleus central to arousal regulation [107, 108]. The LC exhibits sexual dimorphism, with females showing greater neuronal density and responsiveness to stress‐related inputs [36, 109], which may bias females toward higher baseline arousal levels. Supporting this, compared to male participants, female participants had a higher HR at baseline (see Supporting Information for data on cardiovascular parameters), consistent with a trait‐level difference in autonomic tone, a physiological proxy of arousal [110]. We did not, however, observe a sex‐by‐time change in HR during tVNS, and an exploratory correlational analysis indicated that the association between heart‐rate level and RT was present under both real and sham stimulation (see Supporting Information). We therefore cannot claim that tVNS induced a sex‐differentiated shift in arousal. We did observe a stimulation‐specific signal: under real but not sham tVNS, a larger within‐session reduction in HR was associated with greater precision (lower PE). Hence, we propose that pre‐existing differences in baseline arousal may have shaped how tVNS affected behavior, an account that resonates with the Yerkes–Dodson law, which predicts an inverted U‐shaped relationship between arousal and performance [111]. Female participants, possibly nearer an optimal arousal state, may have been stabilized toward more efficient performance, whereas male participants, with lower baseline arousal, may have been moved away from their optimum. This interpretation remains speculative and is consistent with, but not demonstrated by, the present physiological data. Moreover, this speculative interpretation is consistent with the literature suggesting that neurons of the LC fire tonically at low frequencies in under‐aroused states and excessively in hyper‐aroused ones, with phasic firing patterns most conducive to focused attention and efficient behavior [112, 113].

## 6. Conclusion and Limitations

In summary, the present findings highlight different sex‐related effects of cerebellar tACS and tVNS on visuomotor precision.

Focusing on the spatial movement metric (PE), we found that tACS modulated performance in a sex‐related manner—enhancing precision for near targets in females and far targets in males. We proposed that the differential impact of tACS may reflect sex‐related variations in motor planning strategies, with females potentially relying more on fine‐grained control and males favoring spatially guided planning during far‐reaching movements. Thus, we speculated that these differences may be rooted in distinct processing styles, such as variations in visuomotor integration or interhemispheric communication [98], which could vary across sex and influence how stimulation of fronto‐cerebellar circuits alters motor output. Interestingly, the observed improvement in PE and the general increase of RT in both females and males suggest a potential speed–accuracy trade‐off. Specifically, while tACS may have introduced a general interference with motor execution speed, it also appears to have simultaneously modulated sex‐related motor control strategies. In other words, the performance was slower but more accurate, depending on the individual’s underlying motor strategies and, possibly, depending on how these interact with stimulation. Future research is needed to clarify these interaction effects.

In contrast, tVNS did not produce significant stimulation effects on PE, despite showing similar underlying behavioral patterns of the tACS sample. However, for what concerns the time metric, the direction of the tVNS‐related change in RT differed between groups on average—a reduction in the female participants and an increase in the male participants—although the two distributions overlapped substantially (see Figure 4). This pattern points to an influence of sex on preparatory processes, perhaps mediated by differential engagement of the vagus nerve’s influence on attentional and autonomic control systems [42, 44, 105] and sexual dimorphism of the LC [36, 109].

These distinct patterns of results invite interpretive synthesis. Fronto‐cerebellar gamma tACS suggested an action on the sensorimotor processes; it may therefore be best suited to settings in which the goal is to improve sensorimotor control, such as motor (re)learning. tVNS, by contrast, may be best suited when the target is arousal/attentional processes and the study population is one in which the arousal state is a limiting or modifiable factor. These technique‐specific profiles provide an example of what tailoring stimulation to individual factors could mean in practice. For tACS, individualization could extend beyond the frequency tuning employed here to incorporate anatomy—(such as shape and thickness of tissues)—and montage‐related factors that shape the delivered electric field [114]. For tVNS, whose effect here appeared to depend on the arousal state, individualization could involve covarying for baseline autonomic/arousal indices [115] and modulating stimulation intensity and timing to the participant’s position on the arousal–performance curve.

Lastly, methodological limitations must be acknowledged. First, we observed differences in perceptual ratings between real and sham stimulation across both groups (see Supporting Information); however, these differences unlikely accounted for the sex‐related effects, as perceptual discomfort ratings did not vary by sex. Regarding sex‐related effects, a key limitation is the lack of control for hormonal fluctuations in female participants. Failing to confirm menstrual cycle regularity and including women using oral contraceptives precluded us from accounting for potentially relevant factors such as the menstrual cycle phase in the analysis. Given the influence of hormonal changes on synaptic plasticity [22–24] and possibly brain responsiveness to NIBS [21, 25, 26], we cannot exclude that variations in the menstrual cycle phase in our naturally cycling female sample may have contributed to intra‐group variability or modulated the effects of tACS and tVNS. As a result, potential cycle‐dependent influences on cortical excitability and NIBS responsiveness in female participants remain here unaccounted for. Moreover, our sample consisted of young adults of reproductive age. If these stimulation protocols were to be applied in different age groups, such as in children prior to puberty or in older adults, for example, in postmenopausal women, it would be important to consider whether sex‐related effects might differ in relation to age‐related changes in hormonal influences. Future research should therefore examine possible sex‐related differences across developmental and aging stages. Furthermore, sex is an imperfect proxy for the underlying biological and psychosocial mechanisms that may drive the differences we report. Treating sex as a discrete variable necessarily simplifies a continuum of hormonal, anatomical, and experiential factors that vary within, as well as between, groups [116, 117]. In particular, because we did not directly assay circulating hormone levels, our data cannot adjudicate whether the observed differences reflect hormonal, neuroanatomical, or experiential influences; attributing them to any single hormonal mechanism would risk the “estrogen‐therefore‐women” fallacy [117]. We therefore encourage future work that measures the candidate mechanisms (e.g., hormonal status and autonomic tone) directly. Regarding the influence of experiential factors, we also did not record participants’ prior experience with video games or comparable computer‐based tasks, which has been shown to confound apparent sex differences in virtual and computerized motor tasks [82] and could contribute to the near/far differences observed here.

Finally, our effects should be interpreted against the backdrop of the modest and often inconsistent effects of NIBS on motor learning reported in the literature [118]. The real–sham distributions also overlapped substantially in our data, and the sex‐related effects we reported are small relative to inter‐individual variability.

Despite these limitations, our findings underscore that sex is a critical variable in shaping behavioral responses to NIBS and emphasize the importance of tailoring stimulation approaches not only to neural targets and task parameters but also to individual biological factors.

## Funding

This work is supported by the Fondazione Regionale per la Ricerca Biomedica (Regione Lombardia), Project FRRB 3438840 BOOST “Bottom‐up and tOp‐down neuromOdulation of motor plaSTicity in cerebral palsy,” by grants from the Italian Ministry of Health (Ricerca Corrente 2024–2025, Scientific Institute, IRCCS E. Medea to Alessandra Finisguerra).

## Conflicts of Interest

The authors declare no conflicts of interest.

## Endnotes

1

## Supporting Information

Additional supporting information can be found online in the Supporting Information section.

## Supporting information


**Supporting Information** Additional supporting information on EEG preprocessing and analysis on cardiovascular parameters can be found online in the Supporting Information section. Figure S1: Full correlation matrices mapping heart rate (HR) against performance outcomes—reaction time (RT) and precision error (PE)—across the entire active tVNS group and stratified by sex. Figure S2: Ratings of perceptual sensations by group (tACS vs. tVNS) and stimulation condition (real vs. sham). Table S1: Main and interaction effects of the four‐factor model on precision error (PE).

## Data Availability

The datasets generated and analyzed during the study are available from the Open Science Framework, at this link: https://doi.org/10.5281/zenodo.17417281.

## Footnotes

1
^1^In line with current guidance (i.e., Sager Guidelines https://researchintegrityjournal.biomedcentral.com/articles/10.1186/s41073-016-0007-6/tables/1), we use “sex” in a binary categorization as the sex assigned at birth and reported by the participants in response to a 2‐alternative written question.
