# Supplementary material for: Differential Impacts of Fronto‐Cerebellar tACS and tVNS on Motor Learning: Evidence for Sex‐Related Neural Modulation
Source: Neural Plast. 2026 Sep 12;2026:3295986. doi: 10.1155/np/3295986 (PMC13570420; doi:10.1155/np/3295986)
Supplement: Supplementary file 1 [file NP-2026-3295986-s001.docx]

EEG preprocessing for IGF extraction

EEG recordings were acquired using the same tES-EEG device used for tACS delivery (Starstim®, Neuroelectrics, Barcelona, Spain). Signals were sampled at 500 Hz from six Ag/AgCl electrodes (C1, C2, Cz, FC1, FC2, FCz) arranged according to the 10/10 system, with the right earlobe serving as both ground and reference. After acquisition, data were exported to a Matlab®-compatible format (The MathWorks Inc., 2023) and processed using EEGLAB and custom Matlab® scripts. Continuous EEG signals were band-pass filtered between 0.5 and 100 Hz using FIR filters, and a notch filter (48–52 Hz) was applied via the pop_eegfiltnew function in EEGLAB to suppress powerline noise. The data were then re-referenced to the average reference. To extract the individual gamma frequency (IGF), the power spectral density (PSD) was estimated using Welch’s method. The IGF was defined as the frequency with the highest power within the 30–80 Hz gamma range, averaged across all electrodes during the resting-state session.

Analysis on cardiovascular parameters

To assess the safety and potential unwanted effects of the stimulation, heart rate (HR) and oxygen saturation (SpO2) were measures in participants receiving tVNS by using a wrist pulse oximeter (GIMA 34340, GIMA Spa, Italy). Measurements were taken during a 2-minute resting state period at three time points: before tVNS initiation (T0), 30 minutes into stimulation (T1), and 60 minutes after stimulation onset (T2). At each time point, 15 values were recorded, with an 8-second interval between consecutive measurements. Data was analyzed by means of two separate mixed-design 2 × 3 × 2 ANOVA on HR and SpO2, including Stimulation (real vs. sham) and Time (T0, T1, T2) as within-subjects variables, and Sex (females vs. males) as between-subjects variable. The SpO2 of one participant was not recorded due to a technical problem.

Analysis on HR showed a main effect of Sex, F(1,46) = 8.1, p = 0.007, η_p_^2^ = 0.15, indicating higher HR in females (69.38 ± 1.83) than males (62.18 ± 1.75), and a significant main effect of Time, F(2,92) = 8.19, p = 0.005, η_p_^2^ = 0.15, indicating higher HR at T0 (67.39 ± 1.64) as compared to both T1 (65.29 ± 1.21; p = 0.001) and T2 (64.65 ± 1.36; p = 0.001), while the difference between values at T1 and T2 was not significant (p = 0.23). No other effects were significant, all Fs < 2, ps > 0.15.

Analysis on SpO2 revealed a main effect of Sex, F(1,45) = 4.26, p = 0.045, η_p_^2^ = 0.09, indicating higher SpO2 in females (97.69 ± 1.13) than males (97.33 ± 1.12), and a significant interaction of Sex × Time, F(2,90) = 4.98, p = 0.009, η_p_^2^ = 0.10. Post hoc analysis showed increasing SpO2 from T0 (97.45 ± 0.16) to both T1 (97.74 ± 0.15; p = 0.024) and T2 (97.88 ± 0.16; p = 0.024), and from T1 to T2 (p = 0.024) in females only (in males, T0: 97.46 ± 0.16; T1: 97.25 ± 0.13; T2: 97.26 ± 0.15; all ps > 0.3). No other effects were significant, all Fs < 2, ps > 0.23.

Exploratory analyses: heart rate and task performance

To address whether arousal correlates with performance during tVNS, we explored, within the tVNS group, the association between HR (recorded at T0, T1 and T2) and task performance (mean PE and RT), considering both HR levels and within-session HR change scores (Pearson correlations; uncorrected and FDR-corrected p-values).

Two patterns emerged. First, higher HR was associated with slower RT at every time point under real tVNS (T0: r = 0.37; T1: r = 0.39; T2: r = 0.33; all p < .05, FDR-corrected) and with lower PE at T0 (r = −0.34, p = 0.017); however, the HR–RT association was equally present under sham stimulation (T0: r = 0.34, p = 0.017). Second, only under real tVNS, a larger within-session reduction in HR was associated with greater precision: the T2−T0 HR change correlated with PE (r = 0.52, p < 0.001, FDR-corrected; T1−T0: r = 0.38, p = 0.007), an association absent under sham (r = −0.04, p = 0.79) and present in both sexes (males r = 0.48; females r = 0.58). However, when the tVNS-specific effect was isolated through the within-participant real−sham contrast, no association survived (all r ≤ 0.33, p > 0.12, none surviving FDR).

Figure S1. Full correlation matrices mapping Heart Rate (HR) against performance outcomes – Reaction Time (RT) and Precision error (PE) – across the entire active tVNS group and stratified by sex.

Analysis on Stimulation-induced sensations

At the end of each session, participants from both groups completed a questionnaire assessing the intensity of perceptual sensations (itching, pain, burning, heat pain, tingling, metallic taste, fatigue) experienced during the stimulation. Sensations were rated using a 5-point Likert scale, where 0 = “none”, 1 = “mild”, 2 = “moderate”, 3 = “considerable”, and 4 = “strong”. To investigate potential differences in stimulation-induced sensation ratings, we conducted a mixed-design 2 × 2 × 2 ANOVA for each sensation. Group and Sex were included as between-subjects variables, while Stimulation (real vs. sham) was included as within-subjects variable. Figure S1 provides a visual representation of the mean ratings.

The analysis on itching ratings showed a significant main effect of Group, F(1,92) = 29.02, p < 0.0001, η_p_^2^ = 0.24, and Stimulation, F(1,92) = 7.97, p = 0.006, η_p_^2^ = 0.08, further qualified by a significant Group × Stimulation effect, F(1,92) = 9.00, p = 0.004, η_p_^2^ = 0.09, indicating greater itching ratings in active (1.46 ± 0.18) compared to sham stimulation (0.31 ± 0.09; p = 0.0002) during tACS, but not tVNS (active: 0.31 ± ; 0.09 sham: 0.33 ± 0.09; p = 0.9). No other effects were significant, all Fs < 1, ps > 0.57. The model on pain ratings did not yield any significant main or interaction effect, all Fs < 3, ps > 0.14. The model on burning ratings showed a main effect of Group, F(1,92) = 9.76, p = 0.002, η_p_^2^ = 0.1, with higher ratings during tACS (0.87 ± 0.12) than tVNS delivery (0.39 ± 0.09), and Stimulation, F(1,92) = 9.41, p = 0.003, η_p_^2^ = 0.09, with higher ratings during real (0.78 ± 0.09) than sham stimulation (0.48 ± 0.08). No other effects were significant, all Fs < 2, ps > 0.59. The model on heat pain ratings showed a main effect of Group, F(1,92) = 6.75, p = 0.01, η_p_^2^ = 0.07, with higher ratings during tACS (0.84 ± 0.13) than tVNS delivery (0.45 ± 0.09), and Stimulation, F(1,92) = 4.68, p = 0.03, η_p_^2^ = 0.05, with higher ratings during real (0.75 ± 0.09) than sham stimulation (0.54 ± 0.09). No other effects were significant, all Fs < 2, ps > 0.19. The model on tingling ratings yielded a significant main effect of Stimulation, F(1,92) = 15.37, p = 0.0002, η_p_^2^ = 0.14, indicating higher ratings during real (1.26 ± 0.11) than sham stimulation (0.79 ± 0.09). No other effects were significant, all Fs < 4, ps > 0.06. The model on metallic taste ratings did not yield any significant main or interaction effect, all Fs < 3, ps > 0.09. Lastly, the model on fatigue ratings showed a significant main effect of Group, F(1,92) = 6.58, p = 0.01, η_p_^2^ = 0.07, indicating higher ratings in the tACS group (0.28 ± 0.07) than in the tVNS group (0.06 ± 0.05). No other effects were significant, all Fs < 3, ps > 0.12.

Figure S2. Mean ratings of perceptual sensations by Group (tACS vs. tVNS), with color and shape indicating Stimulation condition (real vs. sham). The black dashed line at a rating value of 2 marks the threshold for a “moderate” level of perception. Error bars represent ± 1 SEM. *p < 0.05.

Comparing the perceptual sensations associated with different NIBS techniques is crucial for clinical applications, particularly in populations where tolerability is as important as efficacy, such as in children or other vulnerable groups. The present findings suggest that tACS elicited more pronounced perceptual sensations than tVNS, specifically for itching, burning, heat pain and fatigue. However, it is worth noting that the frequency range, which may modulate perceptual sensations, varied across participants, as it was tailored to their IGF. In contrast, the lower ratings reported for tVNS may reflect the fact that stimulation intensity was calibrated to each participant’s perceptual threshold, potentially minimizing discomfort. These distinctions highlight the importance of considering both the subjective experience and technical parameters of stimulation protocols in trial design.

Tab S1 Main and interaction effects of the Four-factors model on Precision Error (PE)

| **Factors** | **Precision Error** | | | | | | |
| --- | --- | --- | --- | --- | --- | --- | --- |
| **Effect** | | **NumDF** | **DF** | **F** |  | **p-value** | **β** |
| Group | | 1 | 89 | 10.49 |  | 0.002 | -0.06 |
| Distance | | 1 | 53153 | 975.23 |  | < 0.0001 | -0.36 |
| Group × Distance | | 1 | 53153 | 7.80 |  | 0.005 | 0.07 |
| Distance × Sex | | 1 | 53153 | 23.76 |  | < 0.0001 | 0.16 |
| Stimulation × Distance × Sex | | 1 | 53148 | 4.00 |  | 0.046 | -0.15 |
| Group × Stimulation × Distance × Sex | | 1 | 53148 | 6.29 |  | 0.012 | 0.17 |
| All other effects | |  |  | < 4 |  | > 0.054 | ≤ -0.05 |
